# Unusual Giant Right Atrium in Rheumatic Mitral Stenosis and Tricuspid Insufficiency

**DOI:** 10.1155/2011/762873

**Published:** 2011-08-03

**Authors:** Jean Baptiste Anzouan-Kacou, Christophe Konin, Iklo Coulibaly, Roland N'guetta, Anicet Adoubi, Esaïe Soya, Bénédicte Boka

**Affiliations:** ^1^Institut de Cardiologie d'Abidjan, BP V 206 Abidjan, Cote d'Ivoire; ^2^Thorax and Vessels Department, Université de Cocody, 01 BP 166 Abidjan, Cote d'Ivoire; ^3^Université de Bouaké, 27 BP 529 Abidjan 27, Cote d'Ivoire

## Abstract

Dilation and hypertrophy of the atria occur in patients with valvular heart disease especially in mitral regurgitation, mitral stenosis or tricuspid abnormalities. In sub-saharan Africa, rheumatic fever is still the leading cause of valvular heart disease. We report a case of an unusual giant right atrium in context of rheumatic stenosis and severe tricuspid regurgitation in a 58-year-old woman.

## 1. Introduction

Massive right atrial enlargement is common in children, usually owing to rare congenital abnormalities [1]. In adults, it is rare, and the most common causes include valvular heart disease [2]. In sub-Saharan Africa, rheumatic fever is still the leading cause of valvular heart disease [3]. Dilation and hypertrophy of the atria occur in patients with valvular heart disease especially in mitral regurgitation, mitral stenosis or tricuspid abnormalities [4]. We report a case of a giant right atrium in context of rheumatic stenosis.

## 2. Observation

A 58-year-old woman with a history of rheumatic mitral stenosis and atrial fibrillation presented with dyspnea NYHA stage IV, palpitations, and peripheral oedema. These symptoms of progressive heart failure were present for 2 months, but they had worsened during the last 2 days, with increasing dyspnea. She had been diagnosed with a rheumatic mitral stenosis with a mitral valve area of 0.5 cm². At that time, she underwent mitral commissurotomy, but we could not find any others medical information. 

Since then, she had been receiving medical treatment, essentially diuretics, but she had the not seen a cardiologist for many years.

Physical examination revealed that the patient blood pressure was 140/90 mm Hg and her heart rate was 140 b/mn and irregular. The height was 1.5 m, the weight = 52 kg, and the body surface area was 1,5 m². Her jugular veins were distended, and there was congestive hepatomegaly and swollen ankles. Crepitations were present at the basal regions of the lungs. Auscultation revealed a loud S1 at the apex and a loud and spitting S2 at the second left intercostal space, a diastolic rumble at the apex, and a holosystolic murmur at the lower left sternal border. 

**Figure 1:**
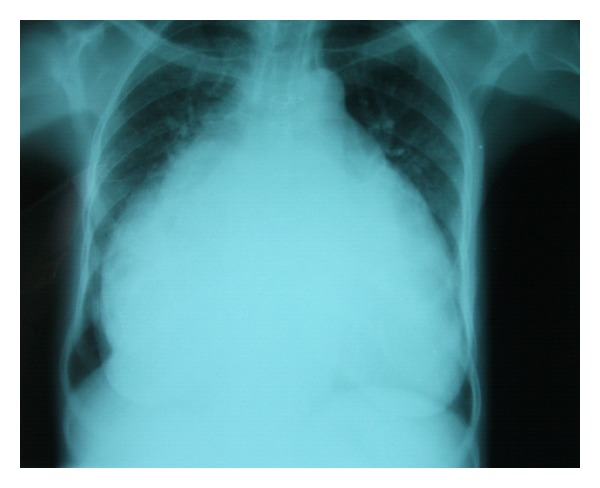
Chest radiography with a marked cardiomegaly suggesting massively dilated right atrium (RA). Cardio thoracic ratio was 89%.

A chest radiography (Figure 1) revealed a marked cardiomegaly suggesting massively dilated right atrium (RA). 

Electrocardiography indicated atrial fibrillation, right axis deviation, incomplete right bundle branch block, and biventricular hypertrophy. 

**Figure 2:**
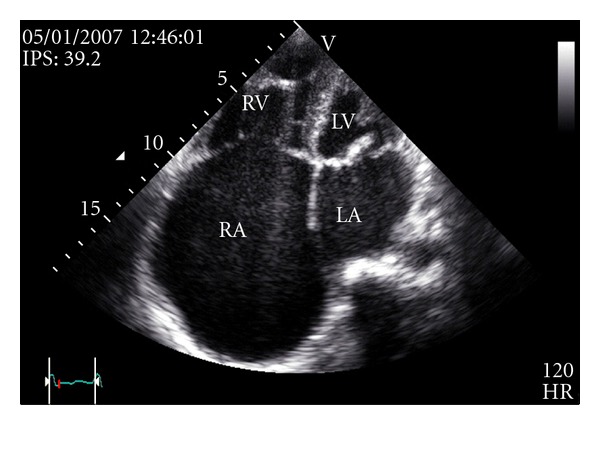
Transthoracic echocardiography, apical four chambers view. Massive enlargement of the right atrium. The right atrial area was 80.6 cm² (53.7 cm²/m²), and the calculated right atrial volume was 621 mL (414 mL/m²). LA: left atrium. LV: left ventricle. RA: right atrium. RV: right ventricle.

Transthoracic echocardiography showed a massive enlargement of the right atrium. The right atrial area was 80.6 cm² (53.7 cm²/m²), and the calculated right atrial volume was 621 mL (414 mL/m²); see Figure 2. 

**Figure 3:**
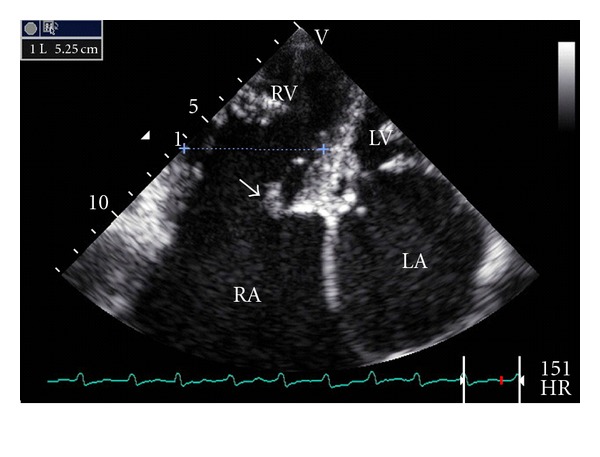
Transthoracic echocardiography, four apical four chambers view. Dilatation of the right ventricle (52.5 mm, mild diameter). The tricuspid valve was thickened and not displaced (white arrow).

The right ventricle was dilated (52.5 mm, mild diameter, apical 4 chambers view, Figure 3). The tricuspid valve was not displaced but was thickened (Figure 3) with restrictive mobility in systole and no coaptation. There was a severe tricuspid regurgitation with a vena contracta width of 0.83 cm and a systolic reversal in hepatic vein flow. 

**Figure 4:**
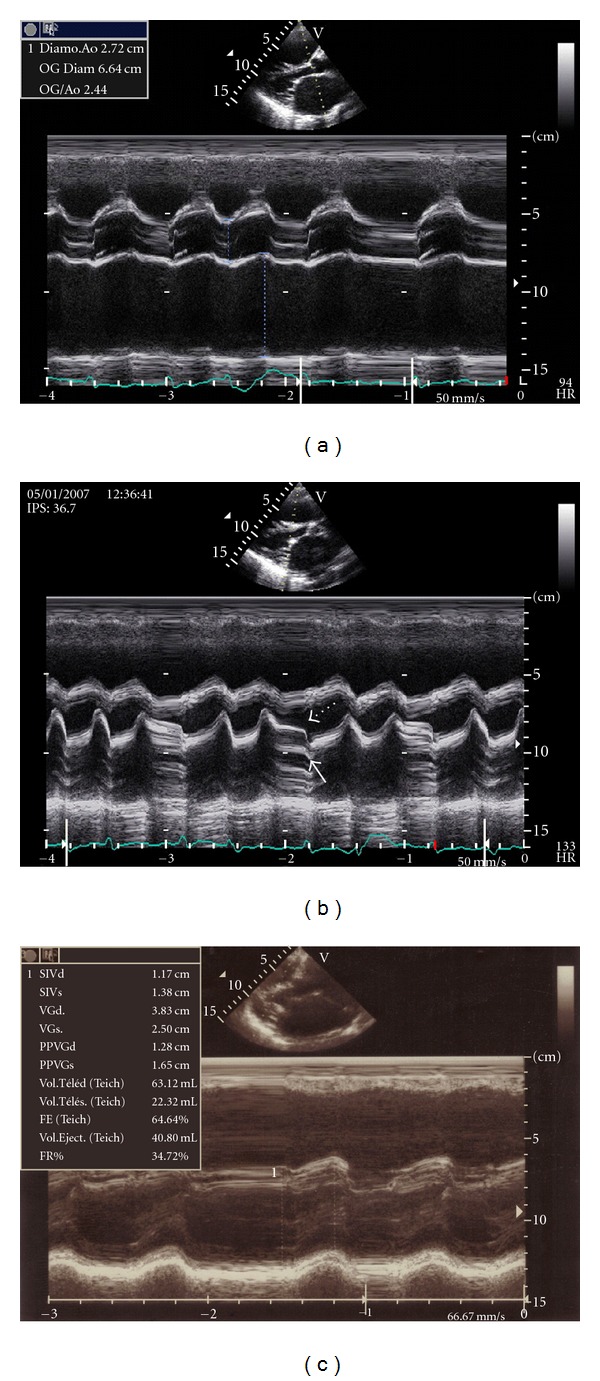
Transthoracic echocardiography, left parasternal long axis view M-mode. (a) dilatation of the left atrium. (b) Paradoxical anterior motion of posterior mitral leaflet (white arrow), with reduced separation of the two leaflets. Reduction of EF slope (broken arrow). (c) Normal left ventricular size, paradoxical septal motion.

The left atrial diameter was 66.4 mm (44.3 mm/m²) (Figure 4(a)) without any thrombus. The mitral valve was thickened with a hockey stick appearance in M mode—Figure 4(b). The planimetered mitral valve area was 1.06 cm² (0.7 cm²/m²) and 0.97 cm² (0.64 cm²/m²) by pressure half-time technique. The mean left atrium-left ventricular diastolic gradient was 7 mm Hg, and the maximal one was 9.9 mm Hg. The left ventricular size was normal (25.5 cm/m²) with a paradoxical septal motion. The ejection fraction was 64% (Figure 4(c)).

We managed and stabilized her with infusions of furosemide and isosorbide dinitrate, digoxin, and spironolacton.

We were not able to obtain her consent for surgery although surgical treatment is available in our institution. She was discharged home after medical stabilization. 

## 3. Discussion

Massive enlargement of the right atrium is usually associated with congenital heart disease in infants and children [5, 6]. In the literature, there are only few cases of giant right atrium in adults [2, 7–9], which must be differentiated from idiopathic right atrial aneurysm [10]. The most common cause of enlarged right atrium in adults are chronic pulmonary disease, severe mitral valvular abnormalities with pulmonary hypertension, pulmonary emboli, and tricuspid valvular abnormalities [7].

Kelesidis et al. [7] reported a giant right atrium with calculated volume of 760 mL in the context of severe tricuspid regurgitation and severe pulmonary hypertension (90 mm Hg) in a 84-year-old woman. Hager et al. [8] reported a case of a 52-year-old man with desmin-related restrictive cardiomyopathy and an estimated right atrial volume of 463 mL.

In our case, the right atrial enlargement may be due to the severe pulmonary hypertension as a consequence of mitral stenosis and severe tricuspid regurgitation. Although we do not have surgical and pathological proof, we think that this regurgitation is a consequence of organic rheumatic tricuspid valve disease. This regurgitation might be also worsened by right ventricular dysfunction and dilatation, persistent pulmonary hypertension, and chronic atrial fibrillation. Mitral valve disease (mitral regurgitation more than mitral stenosis) also leads to giant left atrium [11]. The right atrium size was notably disproportionate to that of the left atrium (see Figure 2). In mitral stenosis, the occurrence of a giant right atrium and a nearly normal-sized or a moderate dilatation of the left atrium has been reported [12]. Calcification of the left atrial myocardium probably the result of organization of intra-atrial thrombus may prevent the left atrium from dilating [12]. In our case, we did not see any calcification in transthoracic echocardiography. Localized pericardial constriction or myocardial fibrosis in left atrium can also be suggested although in our case, we did not have any echocardiographic proof. 

The patient did not underwent surgery although this situation requires a right reduction atrioplasty, tricuspid valve annuloplasty, and mitral valve replacement, after the institution of cardiopulmonary bypass [13]. 

## 4. Conclusion

Despite the poor data and the lack of surgical treatment and followup, we wish to report our findings of a giant right atrium, one of the largest reported to date.
